# Supplementary material for: High-Intensity Interval Training During Cancer Prehabilitation May Improve Cardiorespiratory Fitness: A Meta-Analysis
Source: Healthcare (Basel). 2025 Nov 24;13(23):3030. doi: 10.3390/healthcare13233030 (PMC12692185; doi:10.3390/healthcare13233030)
Supplement: Supplementary file 1 [file healthcare-13-03030-s001.zip › healthcare-3952161-supplementary.pdf]

Supplemental digital content

Search strategy

Database searches were conducted, including studies published up to August 1, 2024, to identify relevant literature. Only studies written in English were considered. The searches yielded a total of 176 articles across all databases. The search terms and the number of results retrieved from each individual database are summarized below:

EMBASE (Number of articles: 59)

('neoplasms'/exp OR neoplasms OR 'tumor'/exp OR tumor OR 'neoplasm'/exp OR neoplasm OR 'tumors'/exp OR tumors OR 'neoplasia'/exp OR neoplasia OR neoplasias OR 'cancer'/exp OR cancer OR 'cancers'/exp OR cancers OR 'malignant neoplasm'/exp OR 'malignant neoplasm' OR (malignant AND ('neoplasm'/exp OR neoplasm)) OR 'malignancy'/exp OR malignancy OR 'malignancies'/exp OR malignancies OR 'malignant neoplasms' OR 'neoplasm, malignant' OR 'neoplasms, malignant' OR 'benign neoplasms' OR 'benign neoplasm'/exp OR 'benign neoplasm' OR 'neoplasms, benign' OR 'neoplasm, benig') AND ('exercise, preoperative' OR 'preoperative exercises' OR 'pre-operative conditioning' OR 'conditioning, pre-operative' OR 'pre operative conditioning' OR 'pre-operative conditionings' OR 'pre-operative rehabilitation' OR 'pre operative rehabilitation' OR 'pre-operative rehabilitations' OR 'rehabilitation, pre-operative' OR 'preoperative rehabilitation' OR 'preoperative rehabilitations' OR 'rehabilitation, preoperative' OR 'preoperative conditioning' OR 'conditioning, preoperative' OR 'preoperative conditionings' OR 'pre-operative exercise' OR 'exercise, pre-operative' OR 'pre operative exercise' OR 'pre-operative exercises' OR 'prehabilitation') AND ('high intensity interval training' OR 'high-intensity interval training' OR 'interval training, high-intensity' OR 'interval trainings, high-intensity' OR 'training, high-intensity interval' OR 'trainings, high-intensity interval' OR 'high-intensity intermittent exercise' OR 'exercise, high-intensity intermittent' OR 'exercises, high-intensity intermittent' OR 'high-intensity intermittent exercises' OR 'sprint interval training' OR 'sprint interval trainings')

PUBMED (Number of articles: 37)

((Neoplasms [MeSH Terms] OR Neoplasms OR Tumor OR Neoplasm OR Tumors OR Neoplasia OR Neoplasias OR Cancer OR Cancers OR Malignant Neoplasm OR Malignancy OR Malignancies OR “Malignant Neoplasms” OR “Neoplasm, Malignant” OR “Neoplasms, Malignant” OR “Benign Neoplasms” OR “Benign Neoplasm” OR “Neoplasms, Benign” OR “Neoplasm, Benig”) AND (Preoperative Exercise [MeSH Terms] OR "Exercise, Preoperative" OR "Preoperative Exercises" OR "Pre-operative Conditioning" OR "Conditioning, Pre-operative" OR "Pre operative Conditioning" OR "Pre-operative Conditionings" OR "Pre-operative Rehabilitation" OR "Pre operative Rehabilitation" OR "Pre-operative Rehabilitations" OR "Rehabilitation, Pre-operative" OR "Preoperative Rehabilitation" OR "Preoperative Rehabilitations" OR "Rehabilitation, Preoperative" OR "Preoperative Conditioning" OR "Conditioning, Preoperative" OR "Preoperative Conditionings" OR "Pre-operative Exercise" OR "Exercise, Pre-operative" OR "Pre operative Exercise" OR "Pre-operative Exercises" OR "Prehabilitation") AND (High-Intensity Interval Training [MeSH Terms] OR “High Intensity Interval Training” OR “High-Intensity Interval Trainings” OR “Interval Training, High-Intensity” OR “Interval Trainings, High-Intensity” OR “Training, High-Intensity Interval” OR “Trainings, High-Intensity Interval” OR “High-Intensity Intermittent Exercise” OR “Exercise, High-Intensity Intermittent” OR “Exercises, High-Intensity Intermittent” OR “High-Intensity Intermittent Exercises” OR “Sprint Interval Training” OR “Sprint Interval Trainings”))

SCOPUS (Number of articles: 36)

(TITLE-ABS-KEY (neoplasms OR neoplasms OR tumor OR neoplasm OR tumors
OR neoplasia OR neoplasias OR cancer OR cancers
OR malignant AND neoplasm OR malignancy OR malignancies OR "Malignant
Neoplasms" OR "Neoplasm, Malignant" OR "Neoplasms, Malignant")) AND (TITLE-ABS-KEY
("Preoperative Exercis" OR "Exercise, Preoperative" OR "Preoperative Exercises" OR "Pre-operative
Conditioning" OR "Conditioning, Pre-operative" OR "Pre operative Conditioning" OR "Pre-operative

Conditionings" OR "Pre-operative Rehabilitation" OR "Pre operative Rehabilitation" OR "Pre-operative Rehabilitations" OR "Rehabilitation, Pre-operative" OR "Preoperative Rehabilitation" OR "Preoperative Rehabilitations" OR "Rehabilitation, Preoperative" OR "Preoperative Conditioning" OR "Conditioning, Preoperative" OR "Preoperative Conditionings" OR "Pre-operative Exercise" OR "Exercise, Pre-operative" OR "Pre operative Exercise" OR "Pre-operative Exercises" OR "Prehabilitation")) AND TITLE-ABS-KEY (("High-Intensity Interval Training" OR "High Intensity Interval Training" OR "High-Intensity Interval Trainings" OR "Interval Training, High-Intensity" OR "Interval Trainings, High-Intensity" OR "Training, High-Intensity Interval" OR "Trainings, High-Intensity Interval" OR "High-Intensity Intermittent Exercise" OR "Exercise, High-Intensity Intermittent" OR "Exercises, High-Intensity Intermittent" OR "High-Intensity Intermittent Exercises" OR "Sprint Interval Training" OR "Sprint Interval Trainings"))

WEB OF SCIENCE (Number of articles: 31)

TS=(Prehabilitation OR Preoperative exercise OR “Exercise, Preoperative” OR “Preoperative Exercises” OR “Pre-operative Conditioning” OR “Conditioning, Pre-operative” OR “Pre operative Conditioning” OR “Pre-operative Conditionings” OR “Pre-operative Rehabilitation” OR “Pre operative Rehabilitation” OR “Pre-operative Rehabilitations” OR “Rehabilitation, Pre-operative” OR “Preoperative Rehabilitation” OR “Preoperative Rehabilitations” OR “Rehabilitation, Preoperative” OR “Preoperative Conditioning” OR “Conditioning, Preoperative” OR “Preoperative Conditionings” OR “Pre-operative Exercise” OR “Exercise, Pre-operative” OR “Pre operative Exercise” OR “Pre-operative Exercises” OR “Prehabilitation") AND TS=(High-Intensity Interval Training OR “High Intensity Interval Training” OR “High-Intensity Interval Trainings” OR “Interval Training, High-Intensity” OR “Interval Trainings, High-Intensity” OR “Training, High-Intensity Interval” OR “Trainings, High-Intensity Interval” OR “High-Intensity Intermittent Exercise” OR “Exercise, High-Intensity Intermittent” OR “Exercises, High-Intensity Intermittent” OR “High-Intensity Intermittent Exercises” OR “Sprint Interval Training” OR “Sprint Interval Trainings”) AND TS=(Neoplasms OR Tumor OR Neoplasm OR Tumors OR Neoplasia OR Neoplasias OR Cancer OR Cancers OR Malignant Neoplasm OR Malignancy OR Malignancies OR “Malignant Neoplasms” OR “Neoplasm, Malignant” OR “Neoplasms, Malignant” OR “Benign Neoplasms” OR “Benign Neoplasm” OR “Neoplasms, Benign” OR “Neoplasm, Benig”)

CINAHL (Number of articles: 13)

(Preoperative exercise OR “Exercise, Preoperative” OR “Preoperative Exercises” OR “Pre-operative Conditioning” OR “Conditioning, Pre-operative” OR “Pre operative Conditioning” OR “Pre-operative Conditionings” OR “Pre-operative Rehabilitation” OR “Pre operative Rehabilitation” OR “Pre-operative Rehabilitations” OR “Rehabilitation, Pre-operative” OR “Preoperative Rehabilitation” OR “Preoperative Rehabilitations” OR “Rehabilitation, Preoperative” OR “Preoperative Conditioning” OR “Conditioning, Preoperative” OR “Preoperative Conditionings” OR “Pre-operative Exercise” OR “Exercise, Pre-operative” OR “Pre operative Exercise” OR “Pre-operative Exercises” OR “Prehabilitation”) AND (“High-Intensity Interval Training” OR “High Intensity Interval Training” OR “High-Intensity Interval Trainings” OR “Interval Training, High-Intensity” OR “Interval Trainings, High-Intensity” OR “Training, High-Intensity Interval” OR “Trainings, High-Intensity Interval” OR “High-Intensity Intermittent Exercise” OR “Exercise, High-Intensity Intermittent” OR “Exercises, High-Intensity Intermittent” OR “High-Intensity Intermittent Exercises” OR “Sprint Interval Training” OR “Sprint Interval Trainings”) AND (Neoplasms OR Tumor OR Neoplasm OR Tumors OR Neoplasia OR Neoplasias OR Cancer OR Cancers OR Malignant Neoplasm OR Malignancy OR Malignancies OR “Malignant Neoplasms” OR “Neoplasm, Malignant” OR “Neoplasms, Malignant” OR “Benign Neoplasms” OR “Benign Neoplasm” OR “Neoplasms, Benign” OR “Neoplasm, Benig”)

Supplementary material 2

Consensus on Exercise Reporting Template (CERT)

Authors and Year	Banarjee et al., 2018
Title	Vigorous intensity aerobic interval exercise in bladder cancer patients prior to radical cystectomy: a feasibility randomised controlled trial
Journal	Support Care Cancer
Reviewer and date	SC 13/04/2022

**Item**	**Description**	**Data extraction- details**	**Location**		**Yes: 1 No: 0**	**"Motivation for the attribution of score": eg, "not reported or not described clearly"**
			Principal article	Other		
1	Detailed description of the type equipment used for exercise	Cycle ergometer (Monark 824E; Varberg, Sweden)	Pag.1517, First column		1	
2	Detailed description of qualifications, expertise and/or training	Training sessions were supervised by a small team of exercise science staff	Pag.1516, Second column,		1	
3	Describe whether the exercises are performed individually or in groups.				0	Not decribed clearly
4	Describe whether the exercises are performed with or unsupervised and how they are delivered	Twice-weekly exercise training sessions were supervised	Pag.1516, Second column		1	
5	Detailed description of how it comes adherence was measured and reported exercise program				0	Not described clearly
6	Detailed description of the motivational strategies				0	Not reported
7a	Detailed description of the decision rule(s) to determine the exercise progression	The exercise programme was progressed by gradually adding more load to the flywheel to maintain the target perceived exertion	Pag.1517, First column		0	Not described clearly
7b	Detailed description of how the exercise program was progressed				0	Not described clearly
8	Detailed description of each exercise to enable replication	Following a 5–10-min warm-up against light resistance (50 W), the aim was for patients to perform 6 × 5 min intervals at a target perceived exertion	Pag.1517, First column		1	

		of 13–15 (‘somewhat hard’ to ‘hard’, equating to 70–85% predicted maximum heart rate based on 220-age), with 2.5 min interpolated active rest intervals against light resistance (50 W). They were instructed to maintain a steady pedalling cadence of 50– 60 rev min−1 during the aerobic intervals Immediately following the aerobic intervals, patients performed a ‘cool-down’ against low resistance (50 W)				
9	Detailed description of any home programme component				0	Not reported
10	Describe whether there are any non-exercise components				0	Not reported
11	Describe the type and number of adverse events that occur during exercise	There were no adverse events resulting from the supervised exercise session	Pag.1518, Second column		1	
12	Describe the setting in which the exercises are performed	Exercise facility at the University of East Anglia, UK, which is close to the treating hospital	Pag.1517, First column		1	
13	Detailed description of the exercise intervention	Following a 5–10-min warm-up against light resistance (50 W), the aim was for patients to perform 6 × 5 min intervals at a target perceived exertion of 13–15 (‘somewhat hard’ to ‘hard’, equating to 70–85% predicted maximum heart rate based on 220-age), with 2.5 min interpolated active rest intervals against light resistance (50 W). They were instructed to maintain a steady pedalling cadence of 50– 60 rev min−1 during the aerobic intervals Immediately following the aerobic intervals, patients performed a ‘cool-down’ against low resistance (50 W)	Pag.1517, First column		1	
14a	Describe whether the exercises are generic (one size fits all) or tailored	The aim was for patients to perform 6 × 5 min intervals at a target perceived exertion of 13–15 (‘somewhat hard’ to ‘hard’, equating to 70–85% predicted maximum heart rate based on 220-age)	Pag.1517, First column		1	
14b	Detailed description of how exercises are tailored to the individual				1	
15	Describe the decision rule for determining the starting level	The Borg Ratings of Perceived Exertion (RPE) Scale to control intensity - 6 × 5 min	Pag.1517, First column		1	

		intervals at a target perceived exertion of 13–15 (‘somewhat hard’ to ‘hard’, equating to 70–85% predicted maximum heart rate based on 220-age)				
16a	Describe how adherence or fidelity is assessed/measured				0	Not reported
16b	Describe the extent to which the intervention was delivered as planned	The median number of supervised exercise sessions attended by patients in the exercise arm was 8 (range 1–10) over a pre-operative period of 3–6 weeks	Pag.1518, Second column		1	

Authors and Year					Blackwell et al., 2020	
Title					High-intensity interval training produces a significant improvement in fitness in less than 31 days before surgery for urological cancer: a randomised control trial	
Journal					Prostate Cancer and Prostatic Diseases	
Reviewer and date					SC 17/04/2022	
**Item**	**Description**	**Data extraction- details**	**Location**		**Yes: 1 No: 0**	**“Motivation for the attribution of score”: eg, “not reported or not described clearly"**
			Principal article	Other		
1	Detailed description of the type equipment used for exercise	Cycle ergometer	Pag. 698, First column		1	
2	Detailed description of qualifications, expertise and/or training	The HIIT sessions were delivered on an individual basis at a university exercise laboratory, fully supervised by a medically qualified doctor	Pag. 698, First column		1	
3	Describe whether the exercises are performed individually or in groups.	The HIIT sessions were delivered on an individual basis	Pag. 698, First column		1	
4	Describe whether the exercises are performed with or unsupervised and how they are delivered	The HIIT sessions were delivered on an individual basis at a university exercise laboratory, fully supervised	Pag. 698, First column		1	
5	Detailed description of how it comes adherence was measured and reported exercise program	Adherence to the intervention was defined as completing at least ten HIIT sessions.	Pag.697, Second column		1	
6	Detailed description of the motivational strategies				0	Not reported
7a	Detailed description of the decision rule(s) to determine the exercise	An increase in wattage was implemented at the mid-way point of training to maintain	Pag. 698, First column		1	

	progression	exercise intensity with progression				
7b	Detailed description of how the exercise program was progressed				1	
8	Detailed description of each exercise to enable replication	The HIIT protocol was performed on a cycle ergometer and comprised a 2-min warm-up period of unloaded cycling, followed by 5, 1-min exertions at 100–115% of the maximal load (watts (W)) reached during their initial CPET, ending with a 2-min recovery period of unloaded cycling	Pag. 698, First column		1	
9	Detailed description of any home programme component	Both HIIT and CON groups were instructed to maintain their habitual physical activity and dietary regimes for the duration of the study	Pag. 698, First column		1	
10	Describe whether there are any non-exercise components				0	Not reported
11	Describe the type and number of adverse events that occur during exercise	There were no adverse safety events reported throughout the study	Pag. 699 Second column		1	
12	Describe the setting in which the exercises are performed	The HIIT sessions were delivered on an individual basis at a university exercise laboratory	Pag. 698, First column		1	
13	Detailed description of the exercise intervention	The HIIT protocol was performed on a cycle ergometer and comprised a 2-min warm-up period of unloaded cycling, followed by 5, 1-min exertions at 100–115% of the maximal load (watts (W)) reached during their initial CPET, ending with a 2-min recovery period of unloaded cycling	Pag. 698, First column		1	
14a	Describe whether the exercises are generic (one size fits all) or tailored	1-min exertions at 100–115% of the maximal load (watts (W)) reached during their initial CPET, ending with a 2-min recovery period of unloaded cycling	Pag. 698, First column		1	
14b	Detailed description of how exercises are tailored to the individual				1	
15	Describe the decision rule for determining the starting level	Five, 1-min exertions at 100–115% of the maximal load (watts (W)) reached during their initial CPET	Pag. 698, First column		1	
16a	Describe how adherence or fidelity is assessed/measured				0	Not reported
16b	Describe the extent to which the intervention	For those patients randomised to HIIT,	Pag.698, Second		1	

	was delivered as planned	adherence (ten or more HIIT sessions) to the exercise training protocol was 84% (16/19)	column			

**Authors and Year**	Dunne et al., 2016
**Title**	Randomized clinical trial of prehabilitation before planned liver resection
**Journal**	The British Journal of Surgery
**Reviewer and date**	SC 17/04/2022

**Item**	**Description**	**Data extraction- details**	**Location**		**Yes: 1 No: 0**	**"Motivation for the attribution of score": eg, "not reported or not described clearly"**
			Principal article	Other		
1	Detailed description of the type equipment used for exercise	Cycle ergometer (Monark 824E; Varberg, Sweden)	Pag.505, Second column		1	
2	Detailed description of qualifications, expertise and/or training	Feasibility of a 4-week supervised preoperative exercise programme	Pag.505, first column		0	
3	Describe whether the exercises are performed individually or in groups.				0	Not decribed clearly
4	Describe whether the exercises are performed with or unsupervised and how they are delivered	Supervised preoperative exercise programme.	Pag.505, Second column		1	
5	Detailed description of how it comes adherence was measured and reported exercise program				0	Not decribed clearly
6	Detailed description of the motivational strategies				0	Not reported
7a	Detailed description of the decision rule(s) to determine the exercise progression				0	Not reported
7b	Detailed description of how the exercise program was progressed				0	Not reported
8	Detailed description of each exercise to enable replication	Prehabilitation consisted of 12 interval exercise sessions over a 4-week period. The programme was developed within an exercise laboratory, and validated in a healthy population. Two recovery exercise sessions were included at the end of the first and fourth weeks (sessions 3 and 12). The interval sessions included a warm-up and warm-down, and 30 min of	Pag.505, Second column		1	

		interval training alternating between exercise of moderate (less than 60 per cent VO_2 at peak exercise) and vigorous (more than 90 per cent VO_2 at peak) intensity				
9	Detailed description of any home programme component				0	Not reported
10	Describe whether there are any non-exercise components				0	Not reported
11	Describe the type and number of adverse events that occur during exercise	There were no reported adverse outcomes of the exercise intervention.	Pag. 509, First column		1	
12	Describe the setting in which the exercises are performed	The programme was developed within an exercise laboratory	Pag.505, Second column		1	
13	Detailed description of the exercise intervention	Prehabilitation consisted of 12 interval exercise sessions over a 4-week period. The programme was developed within an exercise laboratory, and validated in a healthy population. Two recovery exercise sessions were included at the end of the first and fourth weeks (sessions 3 and 12). The interval sessions included a warm-up and warm-down, and 30 min of interval training alternating between exercise of moderate (less than 60 per cent VO_2 at peak exercise) and vigorous (more than 90 per cent VO_2 at peak) intensity	Pag.505, Second column		1	
14a	Describe whether the exercises are generic (one size fits all) or tailored	The exercise programme was personalized to candidates following a standardized equation based on the work rate at their anaerobic threshold on baseline CPET	Pag.505, Second column		1	
14b	Detailed description of how exercises are tailored to the individual				1	
15	Describe the decision rule for determining the starting level	30 min of interval training alternating between exercise of moderate (less than 60 per cent VO_2 at peak exercise) and vigorous (more than 90 per cent VO_2 at peak) intensity	Pag.505, Second column		1	
16a	Describe how adherence or fidelity is assessed/measured	18 of 19 completed 100% of the exercise sessions, with one patient missing two sessions whilst having	Pag.509, First column		1	

		emergency colonic stenting for an obstructing primary tumour				
16b	Describe the extent to which the intervention was delivered as planned				0	Not decribed clearly

Authors and Year			Djurhuus et al., 2023			
Title			Exercise training to increase tumour natural killer-cell infiltration in men with localised prostate cancer: a randomised controlled trial			
Journal			BJU International			
Reviewer and date			SC 17/04/2022			
**Item**	**Description**	**Data extraction- details**	**Location**		**Yes: 1 No: 0**	**"Motivation for the attribution of score": eg, "not reported or not described clearly"**
			Principal article	Other		
1	Detailed description of the type equipment used for exercise	Stationary bicycle ergometer	Pag. 117, Second column		1	
2	Detailed description of qualifications, expertise and/or training	Each session will be supervised by trained instructors		Supp material	0	Not described clearly
3	Describe whether the exercises are performed individually or in groups.				0	Not reported
4	Describe whether the exercises are performed with or unsupervised and how they are delivered	Each session will be supervised by trained instructors		Supp material	1	
5	Detailed description of how it comes adherence was measured and reported exercise program	Overall, 55% (11/20) of the participants attended ≥75% of the exercise sessions during a minimum of 5 weeks	Pag. 119, First column		1	
6	Detailed description of the motivational strategies				0	Not reported
7a	Detailed description of the decision rule(s) to determine the exercise progression	The exercise program consisted of 4 periods where period 1 (week 1) was an adaptation period composed of 4 cycles of HI at 100% Wpeak. Period 2 (week 2) and period 3 (week 3+4) consisted of 4 and 5 cycles of HI at 110% and 120% Wpeak, respectively. Lastly, period 4 (week 5 to 8) comprised six cycles of HI at 120% Wpeak. Trained instructors supervised each session to ensure proper technique and progression in training load		Supp material	1	
7b	Detailed description of how the exercise program was progressed			Supp material	1	
8	Detailed description of each exercise to enable replication	The exercise program consisted of 4 periods where period 1 (week 1) was an adaptation period composed of 4 cycles of HI at 100% Wpeak. Period 2 (week 2) and period 3 (week 3+4) consisted of 4 and 5 cycles of		Supp material	1	

		HI at 110% and 120% Wpeak, respectively. Lastly, period 4 (week 5 to 8) comprised six cycles of HI at 120% Wpeak. Trained instructors supervised each session to ensure proper technique and progression in training load 10 min warm-up performed at 30% of peak power output (Wpeak) determined in the maximal incremental exercise test. The warm-up was followed by 20-25 min of aerobic HIIT consisting of cycles of high-intensity intervals (HI) for 1 min, followed by 3 min of recovery at 30% Wpeak				
9	Detailed description of any home programme component	Participants in the control group were instructed to maintain their everyday lifestyle, including physical activity, during their participation in the study	Pag. 117, Second column		1	
10	Describe whether there are any non-exercise components				0	Not reported
11	Describe the type and number of adverse events that occur during exercise	No adverse events related to exercise were observed	Pag. 119, First column		1	
12	Describe the setting in which the exercises are performed	This was a prospective, RCT based at the Centre for Physical Activity Research at Rigshospitalet, Copenhagen, Denmark			0	Not described clearly (Is not weel defined if is the location where exercise group attended the intervention)
13	Detailed description of the exercise intervention	The exercise program consisted of 4 periods where period 1 (week 1) was an adaptation period composed of 4 cycles of HI at 100% Wpeak. Period 2 (week 2) and period 3 (week 3+4) consisted of 4 and 5 cycles of HI at 110% and 120% Wpeak, respectively. Lastly, period 4 (week 5 to 8) comprised six cycles of HI at 120% Wpeak. Trained instructors supervised each session to ensure proper technique and progression in training load 10 min warm-up performed at 30% of peak power output (Wpeak) determined in the maximal incremental exercise test. The warm-up was followed by 20-25 min of		Supp material	1	

		aerobic HIIT consisting of cycles of high-intensity intervals (HI) for 1 min, followed by 3 min of recovery at 30% Wpeak				
14a	Describe whether the exercises are generic (one size fits all) or tailored	The exercise intervention ranged from 2 to 8 weeks, depending on scheduled RP. The HIIT consisted of 4–6 cycles of highintensity intervals for 1 min at 100–120% of peak power output (Wpeak), followed by 3 min of active recovery at 30% of Wpeak. The exercise programme consisted of four periods comprising week 1 (period 1), week 2 (period 2), weeks 3 + 4 (period 3), and weeks 5–8 (period 4). The number of cycles and intensity increased during each period		Supp material	1	
14b	Detailed description of how exercises are tailored to the individual				1	
15	Describe the decision rule for determining the starting level	Peak power output (Wpeak) determined in the maximal incremental exercise test. 20-25 min of aerobic HIIT consisting of cycles of high-intensity intervals (HI) for 1 min, followed by 3 min of recovery at 30% Wpeak		Supp material	1	
16a	Describe how adherence or fidelity is assessed/measured				0	Not Reported
16b	Describe the extent to which the intervention was delivered as planned	In the protocol intervention period was eight weeks for all participants, and per-protocol analysis was defined as completed ≥75% of the exercise sessions in the intervention period. We changed the per-protocol definition to completion of ≥75% of the exercise sessions during a minimum of five weeks (study week 1 to 5)		Supp material	1	

Authors and Year	Licker et al., 2017
Title	Short-Term Preoperative High-Intensity Interval Training in Patients Awaiting Lung Cancer Surgery: A Randomized Controlled Trial
Journal	Journal of Thoracic Oncology
Reviewer and date	SC 17/04/2022

**Item**	**Description**	**Data extraction- details**	**Location**		**Yes: 1 No: 0**	**“Motivation for the attribution of score”: eg, “not reported or not described clearly"**
			Principal article	Other		
1	Detailed description of the type equipment used for exercise	Rehab group exercised on a cycling ergometer	Pag. 324, Second column		1	
2	Detailed description of qualifications, expertise and/or training	Under the supervision of physiotherapists	Pag. 324, Second column		1	
3	Describe whether the exercises are performed individually or in groups.				0	Not reported
4	Describe whether the exercises are performed with or unsupervised and how they are delivered	Participants in the Rehab group exercised on a cycling ergometer in the outpatient clinic two to three times a week under the supervision of physiotherapists	Pag. 324, Second column		1	
5	Detailed description of how it comes adherence was measured and reported exercise program	Adherence to the HIIT program was defined as the ratio of the number of attended sessions to the number prescribed	Pag. 327, First column		1	
6	Detailed description of the motivational strategies				0	Not reported
7a	Detailed description of the decision rule(s) to determine the exercise progression	The work rate was adjusted by the physiotherapist during each session to target near-maximal heart rates toward the end of each series of sprints on the basis of the individual’s exercise response	Pag. 324, Second column		1	
7b	Detailed description of how the exercise program was progressed				1	
8	Detailed description of each exercise to enable replication	After a 5-minute warm-up period at 50% at peak work rate (peakWR) the patients completed two 10-minute series of 15-second sprint intervals (at 80%–100% peakWR) interspersed by 15-second pauses and a 4-minute rest between the two series. The patients then cooled down with a 5-minute active recovery period at 30% peakWR.<br>Additional exercises, such as	Pag. 324, Second column		1	

		leg press, leg extension, back extension, seat row, biceps curls, or chest and shoulder press, were proposed on an individual basis				
9	Detailed description of any home programme component	Patients in the two groups were given advice regarding active mobilization (at least four 30-minute walks per week)	Pag. 324, Second column		1	
10	Describe whether there are any non-exercise components	Patients in the two groups were given advice regarding active mobilization (at least four 30-minute walks per week) and risk factor management (e.g., healthy nutrition and smoking and alcohol cessation).	Pag. 324, Second column		1	
11	Describe the type and number of adverse events that occur during exercise	No serious adverse events was reported during the HIIT sessions	Pag. 327 First column		1	
12	Describe the setting in which the exercises are performed	Rehab group exercised on a cycling ergometer in the outpatient clinic two to three times a week	Pag. 324, Second column		1	
13	Detailed description of the exercise intervention	After a 5-minute warm-up period at 50% at peak work rate (peakWR) the patients completed two 10-minute series of 15-second sprint intervals (at 80%–100% peakWR) interspersed by 15-second pauses and a 4-minute rest between the two series. The patients then cooled down with a 5-minute active recovery period at 30% peakWR.	Pag. 324, Second column		1	
14a	Describe whether the exercises are generic (one size fits all) or tailored	The patients completed two 10-minute series of 15-second sprint intervals (at 80%–100% peakWR) interspersed by 15-second pauses and a 4-minute rest between the two series	Pag. 324, Second column		1	
14b	Detailed description of how exercises are tailored to the individual				1	
15	Describe the decision rule for determining the starting level	The patients completed two 10-minute series of 15-second sprint intervals (at 80%–100% peakWR).	Pag. 324, Second column		1	
16a	Describe how adherence or fidelity is assessed/measured				0	Not reported
16b	Describe the extent to which the intervention was delivered as planned	Adherence to the prescribed training sessions was 87 ± 18% (a median of eight sessions [IQR25%–75% =7–10])			1	

Authors and Year	West et al., 2015
Title	Effect of prehabilitation on objectively measured physical fitness after neoadjuvant treatment in preoperative rectal cancer patients: a blinded interventional pilot study
Journal	British Journal of Anaesthesia
Reviewer and date	SC 17/04/2022

**Item**	**Description**	**Data extraction- details**	**Location**		**Yes: 1 No: 0**	**"Motivation for the attribution of score": eg, "not reported or not described clearly"**
			Principal article	Other		
1	Detailed description of the type equipment used for exercise	Interval training on an electromagnetically braked cycle ergometer (Optibike Ergoline GmbH, Germany)	Pag. 245, Second column		1	
2	Detailed description of qualifications, expertise and/or training				0	Not Reported
3	Describe whether the exercises are performed individually or in groups.	All subjects exercised in pairs for camaraderie	Pag. 245, Second column		1	
4	Describe whether the exercises are performed with or unsupervised and how they are delivered	Attended a 6-week supervised in-hospital exercise training programme	Pag. 245, Second column		1	
5	Detailed description of how it comes adherence was measured and reported exercise program	Percentage of the 18 sessions completed	Pag. 248, First column		1	
6	Detailed description of the motivational strategies				0	Not reported
7a	Detailed description of the decision rule(s) to determine the exercise progression	The interval-training programme consisted of alternating moderate (80% of work rate at VO_2 at anaerobic threshold by 3 min intervals) to severe (50% of the difference in work rates between VO_2 at peak and VO_2 at anaerobic threshold by 2 min intervals) intensities (total 20 min) for the first two sessions. This is then increased to 40 min (6×3 min intervals at moderate intensity and 6×2 min intervals at severe intensity)	Pag. 245, Second column		1	
7b	Detailed description of how the exercise program was progressed				1	
8	Detailed description of each exercise to enable replication	The interval-training programme consisted of alternating moderate (80% of work rate at VO_2 at anaerobic threshold by 3 min intervals) to severe (50% of the	Pag. 245, Second column		1	

		difference in work rates between VO_2 at peak and VO_2 at anaerobic threshold by 2 min intervals) intensities (total 20 min) for the first two sessions. This is then increased to 40 min (6×3 min intervals at moderate intensity and 6×2 min intervals at severe intensity)				
9	Detailed description of any home programme component				0	Not reported
10	Describe whether there are any non-exercise components				0	Not reported
11	Describe the type and number of adverse events that occur during exercise	No adverse events	Pag. 249, First column		1	
12	Describe the setting in which the exercises are performed	In-hospital exercise training programme	Pag.245 Second column		1	
13	Detailed description of the exercise intervention	The interval-training programme consisted of alternating moderate (80% of work rate at VO_2 at anaerobic threshold by 3 min intervals) to severe (50% of the difference in work rates between VO_2 at peak and VO_2 at anaerobic threshold by 2 min intervals) intensities (total 20 min) for the first two sessions. This is then increased to 40 min (6×3 min intervals at moderate intensity and 6×2 min intervals at severe intensity)	Pag.245 Second column		1	
14a	Describe whether the exercises are generic (one size fits all) or tailored	The training programme was modified for each individual's ramped CPET protocol results ensuring consistent and individualized intensities for all subjects	Pag.245 Second column		1	
14b	Detailed description of how exercises are tailored to the individual				1	
15	Describe the decision rule for determining the starting level	The interval-training programme consisted of alternating moderate (80% of work rate at VO_2 at anaerobic threshold by 3 min intervals) to severe (50% of the difference in work rates between VO_2 at peak and VO_2 at anaerobic threshold by 2 min intervals) intensities (total 20 min) for the first two sessions.	Pag.245 Second column		1	
16a	Describe how adherence				0	Not Reported

	or fidelity is assessed/measured					
16b	Describe the extent to which the intervention was delivered as planned	Percentage of the 18 sessions completed) was 96 (5)%	Pag.248<br><br>First column		1	

Authors and Year	Wood et al., 2020
Title	Lessons learned from a pilot randomized clinical trial of homebased exercise prescription before allogeneic hematopoietic cell transplantation
Journal	Supportive Care in Cancer
Reviewer and date	SC 17/04/2022

**Item**	**Description**	**Data extraction- details**	**Location**		**Yes: 1 No: 0**	**"Motivation for the attribution of score": eg, "not reported or not described clearly"**
			Principal article	Other		
1	Detailed description of the type equipment used for exercise	Participants were individually counseled to discuss available local training resources and were encouraged to choose one or more modes of exercise (from a list that included walking, jogging, running, cycling, elliptical, or stair climbing) for IET sessions. Participants were directed to observe their heart rates on the FitBit Surge during IET sessions in order to achieve pre-defined and programmed target heart rates, calculated for each participant as 80% of the maximum heart rate (80% MHR) as determined by the CPET.	Pag. 3		0	Not reported clearly
2	Detailed description of qualifications, expertise and/or training				0	Not reported clearly
3	Describe whether the exercises are performed individually or in groups.	Individually counseled to discuss available local training resources and were encouraged to choose one or more modes of exercise (from a list that included walking, jogging, running, cycling, elliptical, or stair climbing) for IET sessions. Participants were directed to observe their heart rates on the FitBit Surge during IET sessions in order to achieve pre-defined and programmed target heart rates, calculated for each participant as 80% of the maximum heart rate (80% MHR) as determined by the CPET.	Pag.3		0	Not reported clearly
4	Describe whether the exercises are performed	Individually counseled to discuss available local training	Pag. 3		1	

	with or unsupervised and how they are delivered	resources and were encouraged to choose one or more modes of exercise (from a list that included walking, jogging, running, cycling, elliptical, or stair climbing) for IET sessions. Participants were directed to observe their heart rates on the FitBit Surge during IET sessions in order to achieve pre-defined and programmed target heart rates, calculated for each participant as 80% of the maximum heart rate (80% MHR) as determined by the CPET.				
5	Detailed description of how it comes adherence was measured and reported exercise program				0	Not reported clearly
6	Detailed description of the motivational strategies	Participants were also provided with weekly motivational phone calls, and were given personalized step targets on the Fitbit Surge, with a goal of increasing average steps per day by approximately 10% each week	Pag. 4		1	Not reported
7a	Detailed description of the decision rule(s) to determine the exercise progression	During the first week of the intervention period, participants were asked to engage in 30 minutes of walking, jogging and/or running at any intensity for 3-4 days. During weeks two and beyond, participants were asked to perform exercise sessions consisting of a five minutes warm-up followed by five 2-minute intervals targeting 80% MHR or beyond, interspersed with 3-minute bouts of lower-intensity recovery intervals. Each exercise session was designed to be 30 minutes in length.	Pag. 3,		1	
7b	Detailed description of how the exercise program was progressed				1	
8	Detailed description of each exercise to enable replication				0	Not clearly reported
9	Detailed description of any home programme component	During the first week of the intervention period, participants were asked to engage in 30 minutes of walking, jogging and/or running at any intensity for 3-4 days. During weeks two and	Pag. 3		1	

		beyond, participants were asked to perform exercise sessions consisting of a five minutes warm-up followed by five 2-minute intervals targeting 80% MHR or beyond, interspersed with 3-minute bouts of lower-intensity recovery intervals. Each exercise session was designed to be 30 minutes in length. Participants were asked to engage in 3-4 exercise sessions per week during the IET intervention period.				
10	Describe whether there are any non-exercise components				0	Not reported
11	Describe the type and number of adverse events that occur during exercise				0	Not reported
12	Describe the setting in which the exercises are performed				0	Not reported
13	Detailed description of the exercise intervention	During the first week of the intervention period, participants were asked to engage in 30 minutes of walking, jogging and/or running at any intensity for 3-4 days. During weeks two and beyond, participants were asked to perform exercise sessions consisting of a five minute warm-up followed by five 2-minute intervals targeting 80% MHR or beyond, interspersed with 3-minute bouts of lower-intensity recovery intervals. Each exercise session was designed to be 30 minutes in length. Participants were asked to engage in 3-4 exercise sessions per week during the IET intervention period	Pag. 3		1	
14a	Describe whether the exercises are generic (one size fits all) or tailored	During weeks two and beyond, participants were asked to perform exercise sessions of five 2-minute intervals targeting 80% MHR or beyond, interspersed with 3-minute bouts of lower-intensity recovery intervals	Pag.3		1	
14b	Detailed description of how exercises are tailored to the individual				1	
15	Describe the decision rule for determining the starting level	During the first week of the intervention period, participants were asked to			1	

		engage in 30 minutes of walking, jogging and/or running at any intensity for 3-4 days. During weeks two and beyond, participants were asked to perform exercise sessions consisting of a five minute warm-up followed by five 2-minute intervals targeting 80% MHR or beyond, interspersed with 3-minute bouts of lower-intensity recovery intervals				
16a	Describe how adherence or fidelity is assessed/measured				0	Not reported
16b	Describe the extent to which the intervention was delivered as planned				0	Not clearly reported
